# Safety and Efficacy of the Noncessation Method of Antithrombotic Agents after Emergency Endoscopic Hemostasis in Patients with Nonvariceal Upper Gastrointestinal Bleeding: A Multicenter Pilot Study

**DOI:** 10.1155/2021/6672440

**Published:** 2021-05-22

**Authors:** Daisuke Yamaguchi, Naoyuki Tominaga, Koichi Miyahara, Nanae Tsuruoka, Yasuhisa Sakata, Yuki Takeuchi, Takuya Matsunaga, Hidenori Hidaka, Takashi Akutagawa, Takahiro Noda, Shinichi Ogata, Seiji Tsunada, Motohiro Esaki

**Affiliations:** ^1^Department of Gastroenterology, National Hospital Organization Ureshino Medical Center, Ureshino, Japan; ^2^Division of Gastroenterology, Department of Internal Medicine, Faculty of Medicine, Saga University, Saga, Japan; ^3^Department of Gastroenterology, Saga-Ken Medical Centre Koseikan, Saga, Japan; ^4^Department of Internal Medicine, Karatsu Red Cross Hospital, Karatsu, Japan

## Abstract

**Background and Aims:**

The present study aimed to clarify the safety and efficacy of the noncessation method of antithrombotic agents after emergency endoscopic hemostasis in patients with nonvariceal upper gastrointestinal bleeding (UGIB).

**Methods:**

In this multicenter, prospective, pilot study, we performed emergency endoscopic hemostasis for nonvariceal UGIB in patients taking antithrombotic agents and resumed the medications without a cessation period (group A). The clinical characteristics, types of antithrombotic agents, UGIB etiology, treatment outcome, and adverse events were evaluated. We used propensity score matching to compare treatment outcomes and adverse events with our previous cohort (group B) in whom antithrombotic agents were transiently discontinued after emergency endoscopic hemostasis.

**Results:**

Forty-three consecutive patients were prospectively enrolled. The main antithrombotic agents were low-dose aspirin and direct oral anticoagulants; 11 patients (25.6%) were taking multiple antithrombotics. Peptic ulcers were the main cause of bleeding (95.4%). Endoscopic hemostasis was successful in all patients and the incidence of rebleeding within a month was 7.0%. Propensity score matching created 40 matched pairs. Endoscopic hemostasis was performed by soft coagulation significantly more frequently in group A than in group B (97.5% versus 60.0%, *P* < 0.001). Neither the rebleeding rate within a month nor thromboembolic event rate was different between the two groups. However, the mean duration of hospitalization was significantly shorter in group A than in group B (8.6 ± 5.2 d versus 14.4 ± 7.1 d, *P* < 0.001).

**Conclusions:**

Antithrombotic agents possibly can be continued after successful emergency endoscopic hemostasis for nonvariceal UGIB.

## 1. Introduction

Nonvariceal upper gastrointestinal bleeding (UGIB) is one of the most common causes of gastrointestinal emergencies, sometimes requiring emergency endoscopic hemostasis [1–7]. This is especially the case with antithrombotic agents [8–10], and such patients usually exhibit more severe clinical signs of nonvariceal UGIB [11]. It therefore seems important to determine optimal clinical management of nonvariceal UGIB, especially in patients requiring emergency endoscopic hemostasis under antithrombotic treatment. Theoretically, antithrombotic agents should be stopped after endoscopic hemostasis considering the risk of recurrent UGIB. However, cessation of antithrombotic agents exposes the patients to the risk of thromboembolic events because of their underlying condition [12]. Such thromboembolic events can occur even in patients having only 5–7 days' discontinuation of the medications [13]. Considering these conflicting risks, the Japan Gastroenterological Endoscopy Society (JGES) revised the endoscopic management guidelines in 2012, which focused more on avoiding thromboembolic events than on GI bleeding adverse events [14]. We previously compared the clinical management of patients with nonvariceal UGIB before and after the revision of the JGES guideline [15] and demonstrated that the cessation period of antithrombotic agents was significantly shortened after the revision of the guideline [11]. However, the risk of rebleeding after emergency endoscopic hemostasis without a cessation period remains uncertain.

The present study aimed (1) to evaluate the safety of the noncessation method of antithrombotic agents after emergency endoscopic hemostasis in patients with nonvariceal UGIB and (2) to compare the clinical outcome and adverse events with our previous cohort in whom antithrombotic agents were transiently discontinued after emergency endoscopic hemostasis.

## 2. Methods

### 2.1. Study Design and Ethical Issues

This was designed as a multicenter prospective pilot study. Patients who had undergone emergency endoscopy and endoscopic hemostasis for nonvariceal UGIB from February 2018 through October 2019 at participating institutions were enrolled. Patients above 20 years of age who fulfilled the following criteria were candidates for the study: (1) underwent endoscopic hemostasis within 24 h of symptom onset; (2) used antithrombotic agents, including antiplatelet agents and anticoagulants; and (3) had a clear level of consciousness as well as stable respiratory and circulatory dynamics. Patients with variceal bleeding were excluded. Written informed consent was obtained from patients who met the inclusion criteria after endoscopic hemostasis.

This study was conducted in accordance with the Declaration of Helsinki and the guidelines of the Consolidated Standards of Reporting Trials (CONSORT). The study protocol and the consent procedure were approved by the Ethics Review Committee of the National Hospital Organization Ureshino Medical Center (approval number 17–24) and registered with the University Hospital Medical Information Network (UMIN) Clinical Trials Registry (UMIN000030879) on 22 January 2018.

### 2.2. Patient Data

Clinical data collected in the prospective cohort were age, gender, alcohol consumption, smoking habit, *H. pylori* infection, hemoglobin level, comorbidity (including Charlson comorbidity score), and types of medications at study entry (antithrombotic agents, nonsteroidal anti-inflammatory drugs, and antacids). *H. pylori* infection was diagnosed by the serum levels of anti-*H. pylori* antibodies, the urea breath test, or the rapid urease test. We also accumulated information concerning endoscopic findings, hemostatic procedure, and treatment outcome (transfusion volume, fasting period, and hospitalization period). All patients in the prospective cohort resumed antithrombotic agents after emergency endoscopic hemostasis without a cessation period (group A).

In the present study, we used the clinical data of our retrospective cohort of 154 patients on antithrombotic treatment who underwent emergency endoscopic hemostasis for nonvariceal UGIB from January 2002 through December 2013 at Saga Medical University Hospital as group B (Supplementary Tables 1–3) [11]. These patients were similarly treated but antithrombotic agents were transiently discontinued after endoscopic hemostasis in accordance with the JGES guideline at that time [12, 13].

### 2.3. Endoscopic Hemostasis

Endoscopic hemostasis was performed mainly by high-frequency soft coagulation or hemoclipping using a forward-viewing water-jet endoscope (GIF-Q260J; Olympus, Tokyo, Japan) [16, 17]. The choice of procedure was at the endoscopists' discretion. Soft coagulation was performed using an electrosurgical unit (ICC 200, ERBE Elektronedizin GmbH, Tübingen, Germany) and monopolar hemostatic forceps (Radial Jaw™ 4 Hot Biopsy Forceps, Boston Scientific, Tokyo, Japan) inserted through the channel of the endoscope [16]. Briefly, when the bleeding vessel was identified, electric current was gently applied using the closed forceps to the area surrounding the target vessel using the soft coagulation mode (60 W). Once blood flow diminished, the endoscopist coagulated the target vessel using 80 W in gastric ulcers and 60 W in duodenal ulcers [16]. Hemoclipping was performed using hemoclips (HX-610-135, HX-610-135S hemoclips; Olympus Optical, Tokyo, Japan) that were placed on the ulcer base to bind the detected vessels [16, 18]. In both procedures, when the ulcer base was covered by massive clots, the clots were initially removed either by water jet or by endoscopic forceps to visualize the vessel(s) responsible. Both procedures were repeated until hemostasis was endoscopically confirmed. Interventional radiology and/or surgery was implemented when the endoscopic hemostasis was considered ineffective.

After endoscopic hemostasis was achieved, all patients were hospitalized and managed conventionally (fasting with peripheral parenteral nutrition and intravenous proton pump inhibitors). The indications for blood transfusion were a hemoglobin level <6 g/dL on admission or a rapid drop in hemoglobin level >2 g/dL in patients with hemoglobin level <10 g/dL at baseline. Follow-up endoscopy was performed on patients who were judged necessary by the endoscopist 48–72 h after the initial endoscopy, and repeat endoscopic hemostasis was applied in case recurrent UGIB was detected. Interventional radiology and/or surgery was implemented when the endoscopic hemostasis was considered ineffective. After resumption of oral nutrition, oral administration of the proton pump inhibitors was performed for at least a month in both groups.

All endoscopic hemostasis was performed by 18 endoscopists, comprising 8 specialists and 10 trainees. Specialists were defined as endoscopists who had performed endoscopy for more than 5 years with experience in more than 40 endoscopic submucosal dissection (ESD) procedures after mastering the required fundamental skills and knowledge [19, 20].

### 2.4. Study Outcomes

The primary endpoint of the present study was the rate of rebleeding within a month after emergency endoscopic hemostasis in group A. Rebleeding was defined as follows: (1) follow-up endoscopy identified recurrent UGIB or the stigmata of recent hemorrhage or (2) melena and progressive anemia with a decrease in hemoglobin level greater than 2 g/dL and/or with a decrease in systolic blood pressure <80 mmHg. The secondary endpoint was the incidence of adverse events within a month (rebleeding, thromboembolic events, and mortality) in group A.

To compare the noninferiority of the noncessation method of antithrombotic agents to the transient discontinuation method based on the JGES guidelines, treatment outcome and adverse events in group A were compared with those in group B. Group B was the retrospective cohort of 154 patients with nonvariceal UGIB who underwent emergency endoscopic hemostasis and in whom antithrombotic agents were subsequently discontinued [11].

### 2.5. Sample Size Calculation and Statistical Analysis

The sample size of the noninferiority study was calculated based on the rate of rebleeding within a month. The rebleeding rate after emergency endoscopic hemostasis in patients with nonvariceal UGIB taking antithrombotic agents was 9.7% (15/154 patients) in our previous study [11]. We also considered other studies that described bleeding rates after gastric ESD among patients on antithrombotic treatments (7.0%–21.1%) [21–23]. Based on these results, we assumed the overall rebleeding rate in group A to be 20%. We hypothesized that a difference of 10% or less (prespecified noninferiority margin for this endpoint) in the rebleeding rate between the two groups would constitute a clinically meaningful difference. Assuming a power of 80% and an alpha of 0.05 (one-sided), at least 78 patients (39 patients in each group) would be required in the noninferiority study. Assuming that 10% of patients could be lost to follow-up, a sample size of 86 patients (43 patients in each group) was planned. The rebleeding rate in group A would be considered noninferior to that in group B if the 95% confidence interval (CI) for the treatment difference was greater than −10.0%.

Treatment outcome and adverse events were compared between groups A and B using propensity score matching analysis. This method was applied to adjust significant differences in the baseline characteristics of the patients and reduce the influence of possible confounding factors [24]. The two groups were matched at a 1 : 1 ratio (40 patients in each group) with the adjustment for nine covariates (age, gender, *H. pylori* infection, number of agents [antithrombotic, antiplatelet, and anticoagulant], and ulcer characteristics [location, number, and size]) to minimize inherent bias. These nine covariates were selected based on the opinions of expert endoscopists (DY, NT, KM, TN, SO, and ST). This model yielded a C statistic of 0.705, indicating preferable ability for the comparison between groups A and B. The caliper width of propensity score matching was 0.20.

Categorical data were expressed as a number (percentage), and the chi-square test was used to identify differences between the two groups. Numerical data for a normal distribution were expressed as the mean ± the standard deviation, and Student's *t*-test was used to determine differences between the two groups. Numerical data for a skewed distribution were expressed as median interquartile range, and the Mann–Whitney *U*-test was used. Levels of significance for all comparisons made were reported, whether significant or not, with *P* values or confidence intervals. A *P* value <0.05 was considered to be statistically significant for each test. All statistical analyses were performed with JMP, version 13.0.0 (SAS Institute Inc., Cary, NC, USA).

## 3. Results

### 3.1. Clinical Characteristics of the Prospective Cohort

Forty-three patients who resumed antithrombotic agents without cessation periods were allocated to group A. The characteristics of patients in group A are listed in Table 1. The mean age of the patients was 77.8 years, and 28 patients (65.1%) were males. The rate of *H. pylori* infection was 46.5%. Cardiovascular disease was the most common comorbidity; it had been diagnosed in 23 patients.

Table 2 summarizes the details concerning antithrombotic agents among the patients. Thirty-two patients (74.4%) took a single antithrombotic agent, and 11 patients (25.6%) took multiple antithrombotic agents. The most common antiplatelet agent was aspirin (17 patients), and edoxaban (six patients), one of the direct oral anticoagulants (DOACs), was the most frequently prescribed anticoagulant agent.

### 3.2. Endoscopic Findings and Clinical Outcome of the Prospective Cohort

The endoscopic findings of patients with nonvariceal UGIB are shown in Table 3. Of the 41 patients with peptic ulcer disease, 34 patients complained of bleeding gastric ulcers, among whom multiple gastric ulcers were found in 15 patients. A large ulcer (>11 mm in diameter) was found in 21 patients, and spurting gastroduodenal bleeding was endoscopically identified in seven patients. To achieve endoscopic hemostasis, the soft coagulation method was applied to all but one patient, and 27 procedures (62.8%) were performed by trainees. Consequently, endoscopic hemostasis was successfully carried out in all of the 43 patients.

The mean hemoglobin level on admission was 8.0 ± 2.6 g/dL, and blood transfusion was required with a mean volume of 3.2 units. After endoscopic hemostasis, all patients were treated with fasting and hospitalized for a mean period of 2.2 ± 0.7 d and 9.1 ± 5.5 d, respectively. Rebleeding occurred in three patients (7.0%); however, neither major thromboembolic events nor fatalities were recorded during the follow-up period.

### 3.3. Comparison of Clinical Outcomes between Groups A and B by Propensity Score Matching

Table 4 compares the clinical characteristics between groups A and B before and after propensity score matching. Before propensity score matching, the mean age (77.8 ± 9.1 years in group A versus 72.8 ± 12.4 years in group B, *P* = 0.004), *H. pylori* infection (46.5% versus 69.5%, *P* = 0.007), antiplatelet agents (69.8% versus 86.4%, *P* = 0.02), and anticoagulant agents (39.5% versus 21.4%, *P* = 0.03) were significantly different between the two groups. Propensity score matching subsequently created 40 matched pairs in the present study.

Table 5 compares the treatment outcomes after propensity score matching between the two groups. Because group B comprised a retrospective cohort of patients who transiently discontinued antithrombotic agents after emergency endoscopic hemostasis, the cessation period of antithrombotic agents was significantly shorter in group A than in group B (0 versus 6.5 d; *P* < 0.001). Although the soft coagulation method was more frequently applied in group A than in group B (97.5% versus 60.0%, *P* < 0.001), endoscopic hemostasis was successfully achieved in all patients of both groups. The hospitalization periods were significantly shorter in group A than in group B (8.6 ± 5.2 versus 14.4 ± 7.1 d; *P* < 0.001).

**Figure 1:**
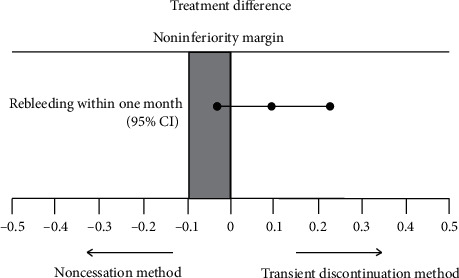
Difference in rebleeding rate within one month in patients taking antithrombotic agents who underwent emergency endoscopic hemostasis for nonvariceal upper gastrointestinal bleeding and resumed antithrombotic medication without a cessation period (group A) or transiently discontinued the medication (group B).

Rebleeding within one month occurred in 2 (5.0%) of 40 patients in group A and 6 (15.0%) of 40 patients in group B. The treatment difference was calculated as 10.0% (95.0% CI: −4.2 to 23.2), within an a priori −10% noninferiority margin. Therefore, the rebleeding risk in the noncessation method of antithrombotic agents after emergency endoscopic hemostasis was not considered to be inferior to the transient discontinuation method in patients with nonvariceal UGIB (Figure 1). Neither thromboembolic events nor fatality occurred during the follow-up period.

## 4. Discussion

In this super-aging society, both antiplatelet and anticoagulant agents have been increasingly used for the prophylaxis and treatment of vascular disease, especially cerebrovascular and cardiovascular disease. However, this practice has also led to an increase in the incidence of and morbidity associated with UGIB [25–28]. In addition, because the combination therapy of antithrombotic agents has become more prevalent [11] and because these medications may be associated with a higher risk of UGIB [29], medical management in patients with UGIB on antithrombotic agents has been a matter of recent debate.

The JGES guideline published in 2012 drastically changed the management of gastroenterological endoscopy in patients undergoing antithrombotic treatment [14]. The guideline was based on the fact that the clinical outcomes of thromboembolic events caused by the transient discontinuation of antithrombotic agents during the periendoscopic period were more severe compared with those of bleeding events among patients treated with the medication [30]. In fact, we also managed two patients who developed severe thromboembolic events after cessation of antithrombotic agents because of UGIB [11]. In contrast, repeat rebleeding in patients with UGIB after endoscopic hemostasis is troublesome for endoscopists, and some reports suggested a possible higher risk of rebleeding in patients with UGIB on antithrombotic agents [31, 32]. Furthermore, the clinical management of antithrombotic agents after invasive gastrointestinal endoscopy remains uncertain. We therefore compared the risk of rebleeding and safety of nonvariceal UGIB between patients on antithrombotic agents with our noncessation method and those treated in accordance with the JGES guidelines.

In the present study, the overall rebleeding rate within a month was 7.0% in patients with nonvariceal UGIB who continued antithrombotic agents after endoscopic hemostasis. The rebleeding rate in our previous cohort, classified as group B in the present study, in whom antithrombotic agents were transiently discontinued, was 9.7% (15/154 patients) [11]. That value was almost equivalent to the rebleeding rates in other previous studies (7.0% to 17.8%) [32–34]. Consequently, the difference in the rebleeding rate between groups A and B after propensity score matching was demonstrated to be within the noninferiority margin. We therefore considered that our noncessation method of antithrombotic agents did not lead to an increased risk of rebleeding after emergency endoscopic hemostasis in patients with nonvariceal UGIB.

The rebleeding rate after endoscopic hemostasis can be influenced by the hemostatic procedures applied. In the present study, the rate of rebleeding was numerically but not significantly higher in group B than in group A after propensity score matching. Because the soft coagulation method is a useful hemostatic procedure during ESD [20, 35–38], ESD has also become more prevalent for endoscopic hemostasis in patients with nonvariceal UGIB in Japan [16, 39]. In addition, because our previous study demonstrated that soft coagulation was significantly superior to hemoclipping for endoscopic hemostasis in such patients [16], we have preferentially chosen the soft coagulation method over hemoclipping. As a result, soft coagulation was more frequently applied in group A than in group B (97.5% in group A versus 60.0% in group B, *P* < 0.001). However, it seems unlikely that the difference in the procedure affected the rebleeding rate because the rebleeding rates by hemoclipping and by soft coagulation were similar in group B (14.3% versus 16.7%, respectively).

No patient experienced major complications (bleeding or thromboembolism) in the present study. However, we previously reported adverse events in two patients who experienced cerebral infarction during the cessation period of antithrombotic agents [11]. Another report also described six thromboembolic events occurring in 36 patients with nonvariceal UGIB who discontinued warfarin as well as one myocardial infarction in 41 patients with nonvariceal UGIB who discontinued aspirin [33]. A meta-analysis also demonstrated that the resumption of warfarin following interruption because of GI bleeding was significantly associated with a reduction in thromboembolic events (hazard ratio [HR]: 0.68, 95% CI: 0.52–0.88) and in mortality (HR: 0.76, 95% CI: 0.66–0.88) [40]. These results indicated that we should be strictly aware of the potential for thromboembolic events by the interruption of antithrombotic agents after UGIB.

In addition, such unfavorable outcome can be also caused by hemodynamic instability due to UGIB. Actually, a cohort study of emergency admissions for nonmalignant UGIB reported that 8.9% of deaths after 30 days of hospitalization were due to ischemic heart disease and the cardiovascular age-standardized mortality rate following UGIB was high [41]. Another study examining the risk of rebleeding and in-hospital mortality after UGIB demonstrated that 1.5% of patients with UGIB died of thromboembolism, and poor performance status (PS; ≥3) and tachycardia (>100/min) were significantly associated with mortality [42]. Considering the higher risk of thromboembolism in patients with UBIG, efforts to decrease the risk seem indispensable, especially in patients with antithrombotic agents. Together with the fact that no major bleeding adverse events occurred in group A of the present study, the noncessation method after emergency endoscopic hemostasis seems to be appropriate for the management of patients with nonvariceal UGIB on antithrombotic treatment.

While no significant difference in rebleeding and thromboembolic adverse events were identified in comparing groups A and B, the hospitalization period was significantly shorter in the former than in the latter. Such a difference might be partly attributed to the numerically higher rate of rebleeding in group B. However, this apparent advantage of the noncessation method of antithrombotic agents after endoscopic hemostasis should be considered cautiously because warfarin requires close monitoring of the prothrombin time and international normalized ratio when resuming. Warfarin was used more frequently as anticoagulant therapy in group B (nine of nine patients, 100%) than in group A (two of eleven patients, 18.2%). Because DOACs have recently become the mainstay of anticoagulant therapy, further study is necessary to determine the difference in hospitalization period among patients with nonvariceal UGIB taking DOACs.

Our present study has some limitations. First, the number of subjects in the prospective cohort was small because of the study design. Therefore, the low rebleeding rate in the prospective cohort can be a statistical type II error. Second, a considerable number of endoscopic hemostases in both groups were performed by trainees. While all procedures of endoscopic hemostasis by trainees were performed under the guidance of specialists, insufficient hemostatic techniques of the trainees could have caused the numerical difference in the rebleeding rate between the two groups. Third, the retrospective cohort of our previous study was used to compare clinical outcome and adverse events between patients with and without a cessation period of antithrombotic agents after endoscopic hemostasis. While propensity score matching analysis was applied to minimize the difference in clinical characteristics between the two groups, the results of the present study should be interpreted with caution because of the different types of cohort between the groups. Further prospective study with a larger number of subjects should be done to validate the results of the present study.

## 5. Conclusions

This multicenter pilot study demonstrated that continuing antithrombotic agents without a cessation period was acceptable after emergency endoscopic hemostasis in patients with nonvariceal UGIB and could shorten the hospitalization period.

## Figures and Tables

**Table 1:** Characteristics of patients in group A^∗^.

Number of patients (N)	43

Age (years)	77.8 ± 9.1

Gender, males	28 (65.1%)

Alcohol drinking	6 (13.9%)

Smoking	16 (37.2%)

*Helicobacter pylori* infection	20 (46.5%)

Using NSAIDs	10 (23.2%)

Using gastric acid secretion inhibitor	11 (25.5%)

Comorbidity	
Cardiovascular diseases	23 (53.5%)
Cerebrovascular diseases	14 (32.6%)
Chronic kidney diseases	8 (18.6%)
Chronic liver damage	2 (4.6%)
Diabetes mellitus	14 (32.6%)
Hypertension	32 (74.4%)
Malignant diseases	10 (23.3%)

Charlson comorbidity score	1.8 ± 1.0

^∗^Results are presented as number of patients or mean ± standard deviation. Group A: patients taking antithrombotic agents who underwent emergency endoscopic hemostasis for nonvariceal upper gastrointestinal bleeding and resumed antithrombotic medication without a cessation period; NSAIDs: nonsteroidal anti-inflammatory drugs.

**Table 2:** Types of antithrombotic agents taken by patients in group A.

Number of antithrombotic agents	43
Single agent	32 (74.4%)
Multiple agents	11 (25.6%)

Types of antithrombotic agents	
Antiplatelet agents	
Aspirin	17 (39.5%)
Clopidogrel	11 (25.6%)
Sarpogrelate	3 (7.0%)
Prostaglandin preparations	2 (4.6%)
Prasugrel	2 (4.6%)
Cilostazol	1 (2.3%)

Anticoagulant agents	
DOACs	12 (27.9%)
Edoxaban	6 (14.0%)
Apixaban	4 (9.3%)
Rivaroxaban	2 (4.6%)
Warfarin	5 (11.6%)

DOACs: direct oral anticoagulants. Group A: patients taking antithrombotic agents who underwent emergency endoscopic hemostasis for nonvariceal upper gastrointestinal bleeding and resumed antithrombotic medication without a cessation period.

**Table 3:** Endoscopic findings and hemostatic methods in patients in group A.

Peptic ulcer	41 (95.4%)
Angioectasia	2 (4.6%)
Location of ulcer	
Gastric ulcer	34 (79.1%)
Upper third	5 (11.6%)
Middle third	23 (53.5%)
Lower third	6 (13.9%)
Duodenal ulcer	7 (16.3%)
Number of ulcers	
Single	26 (60.5%)
Multiple	15 (34.9%)
Size of ulcer (mm)	
0–10	20 (46.5%)
>11	21 (48.1%)
Forrest classification	
Ia	7 (16.3%)
Ib	18 (41.9%)
IIa	18 (41.9%)
Atrophic gastritis	
Closed type	6 (14.0%)
Open type	31 (72.1%)
Hemostatic method	
Soft coagulation	42 (97.7%)
Hemoclips	1 (2.3%)
Operator of hemostasis	
Trainees	27 (62.8%)
Specialists	16 (37.2%)

Group A: patients taking antithrombotic agents who underwent emergency endoscopic hemostasis for nonvariceal upper gastrointestinal bleeding and resumed antithrombotic medication without a cessation period.

**Table 4:** Characteristics of patients before and after propensity score matching in groups A and B.

	Before propensity score matching			
	Group A	Group B	*P* value	Standardized differences
Number of patients (N)	43	154		
Age (years)	77.8 ± 9.1	72.8 ± 12.4	0.004	0.47
Gender, male	28 (65.1%)	108 (70.1%)	0.58	0.11
*Helicobacter pylori* infection	20 (46.5%)	107 (69.5%)	0.007	0.48
Number of antithrombotics, multiple	11 (25.6%)	38 (24.7%)	1.00	0.02
Antiplatelet agents	30 (69.8%)	133 (86.4%)	0.02	0.41
Anticoagulant agents	17 (39.5%)	33 (21.4%)	0.03	0.40
Location of ulcer, gastric	34 (79.1%)	108 (84.4%)	1.00	0.14
Number of ulcers, single	28 (65.1%)	80 (62.5%)	0.86	0.05
Size of ulcer, >11 mm	21 (48.8%)	83 (64.8%)	0.07	0.33

	After propensity score matching			
	Group A	Group B	*P* value	Standardized differences

Number of patients (N)	40	40		
Age (years)	77.1 ± 8.9	78.2 ± 8.0	0.55	0.13
Gender, male	26 (65.0%)	24 (60.0%)	0.82	0.10
*Helicobacter pylori* infection	19 (47, 5%)	20 (50.0%)	1.00	0.05
Number of antithrombotics, multiple	11 (27.5%)	12 (30.0%)	1.00	0.05
Antiplatelet agents	30 (75.0%)	31 (77.5%)	1.00	0.06
Anticoagulant agents	14 (35.0%)	12 (30.0%)	0.81	0.11
Location of ulcer, gastric	33 (82.5%)	36 (90.0%)	0.52	0.22
Number of ulcers, single	26 (65.0%)	26 (65.0%)	1.00	0.00
Size of ulcer, >11 mm	21 (52.5%)	22 (55.0%)	1.00	0.05

Group A: patients taking antithrombotic agents who underwent emergency endoscopic hemostasis for nonvariceal upper gastrointestinal bleeding and resumed antithrombotic medication without a cessation period; group B: patients taking antithrombotic agents who underwent emergency endoscopic hemostasis for nonvariceal upper gastrointestinal bleeding for whom antithrombotic medication was transiently discontinued.

**Table 5:** Comparison of treatment outcome between groups A and B after propensity score matching.

	Group A	Group B	*P* value
Number of patients (N)	40	40	
Cessation period (day)^a^	0 (0–0)	6.5 (3.3–10)	<0.001
Hemoglobin on admission (g/dL)	8.1 ± 2.6	7.6 ± 1.7	0.31
Transfusion volume (U)	3.0 ± 2.6	2.6 ± 2.9	0.58
Endoscopic hemostasis	40 (100%)	40 (100%)	1.00
Main modality of hemostasis			<0.001
Soft coagulation	39 (97.5%)	24 (60.0%)	
Hemoclips	1 (2.5%)	14 (35.0%)	
Absolute ethanol injection	0 (0%)	2 (5%)	
Operator of hemostasis			0.82
Trainees	24 (60.0%)	26 (65.0%)	
Specialists	16 (40.0%)	14 (35.0%)	
Hospitalization periods (day)	8.6 ± 5.2	14.4 ± 7.1	<0.001

^a^Median (interquartile range). Group A: patients taking antithrombotic agents who underwent emergency endoscopic hemostasis for nonvariceal upper gastrointestinal bleeding and resumed antithrombotic medication without a cessation period; group B: patients taking antithrombotic agents who underwent emergency endoscopic hemostasis for nonvariceal upper gastrointestinal bleeding for whom antithrombotic medication was transiently discontinued.

## Data Availability

The datasets used and/or analyzed during the current study are available from the corresponding author upon reasonable request.

## Abbreviations

DOACs: Direct oral anticoagulants
ESD: Endoscopic submucosal dissection
JGES: Japan Gastroenterological Endoscopy Society
UGIB: Upper gastrointestinal bleeding.

## Supplementary Materials

Supplementary Materials Supplementary Table 1: characteristics of patients in group B. Supplementary Table 2: types of antithrombotic agents taken by patients in group B. Supplementary Table 3: endoscopic findings and hemostatic methods in patients in group B.
