# Supplementary material for: Safety and Efficacy of the Noncessation Method of Antithrombotic Agents after Emergency Endoscopic Hemostasis in Patients with Nonvariceal Upper Gastrointestinal Bleeding: A Multicenter Pilot Study
Source: Can J Gastroenterol Hepatol. 2021 May 22;2021:6672440. doi: 10.1155/2021/6672440 (PMC8164533; doi:10.1155/2021/6672440)
Supplement: Supplementary Materials — Supplementary Table 1: characteristics of patients in group B. Supplementary Table 2: types of antithrombotic agents taken by patients in group B. Supplementary Table 3: endoscopic findings and hemostatic methods in patients in group B. [file 6672440.f1.docx]

Suppl.Table 1. Characteristics of patients in group B*

|  |  |
| --- | --- |
| Number of patients (N) | 154 |
| Age (years) | 72.8 ±12.4 |
| Gender, Males | 108 (70.1%) |
| Alcohol drinking | 44 (28.6%) |
| Smoking | 60 (39.0%) |
| *Helicobacter pylori* infection | 91 (71.7%) |
| Using NSAIDs | 33 (21.4%) |
| Using gastric acid secretion inhibitor | 27 (17.5%) |
| Comorbidity |  |
| Cardiovascular diseases | 104 (67.5%) |
| Cerebrovascular diseases | 39 (25.3%) |
| Chronic kidney diseases | 29 (18.8%) |
| Chronic liver damage | 8 (5.2%) |
| Diabetes mellitus | 50 (32.5%) |
| Hypertension | 111 (72.1%) |
| Malignant diseases | 24 (15.6%) |
| Charlson comorbidity score | 1.7 ± 1.0 |

* Results are presented as number of patients or mean ± standard deviation.

Group B: patients taking antithrombotic agents who underwent emergency endoscopic hemostasis for non-variceal upper gastrointestinal bleeding for whom antithrombotic medication was transiently discontinued.

Suppl.Table 2. Types of antithrombotic agents taken by patients in group B

|  |  |
| --- | --- |
| Number of antithrombotic agents | 154 |
| Single agent | 114 (74.0%) |
| Multiple agents | 40 (26.0%) |
| Types of antithrombotic agents |  |
| Antiplatelet agents |  |
| Aspirin | 97 (63.0%) |
| Clopidogrel | 22 (14.3%) |
| Ticlopidine | 15 (9.7%) |
| Cilostazol | 9 (5.8%) |
| Prostaglandin preparations | 8 (5.2%) |
| Sarpogrelate | 2 (1.3%) |
| Eicosapentaenoic acid preparation | 1 (0.6%) |
| Anticoagulant agents |  |
| Warfarin | 37 (24.0%) |

Group B: patients taking antithrombotic agents who underwent emergency endoscopic hemostasis for non-variceal upper gastrointestinal bleeding for whom antithrombotic medication was transiently discontinued.

Suppl.Table3. Endoscopic findings and hemostatic methods in patients in group B

|  |  |
| --- | --- |
| Peptic ulcer | 128 (83.1%) |
| Angioectasia | 11 (7.1%) |
| Mallory–Weiss syndrome | 5 (3.2%) |
| GERD | 5 (3.2%) |
| AGML | 4 (2.6%) |
| GAVE | 1 (0.6%) |
| Location of ulcer |  |
| Gastric ulcer | 107 (69.5%) |
| Upper third | 27 (25.2%) |
| Middle third | 58 (54.2%) |
| Lower third | 22 (13.6%) |
| Duodenal ulcer | 22 (13.6%) |
| Number of ulcer |  |
| Single | 80 (62.5%) |
| Multiple | 48 (37.5%) |
| Size of ulcer (mm) |  |
| 0-10 | 45 (35.2%) |
| >11 | 83 (64.8%) |
| Forrest classification |  |
| Ia | 11 (8.5%) |
| Ib | 53 (41.4%) |
| IIa | 64 (50.0%) |
| Atrophic gastritis |  |
| Closed type | 45 (29.2%) |
| Open type | 72 (46.8%) |
| Hemostatic method |  |
| Soft coagulation | 66 (42.9%) |
| Hemoclips | 52 (33.8%) |
| Operator of hemostasis |  |
| Trainees | 103 (66.9%) |
| Specialists | 51 (33.1%) |

AGML, acute gastric mucosal lesion; GAVE, gastric–antral vascular ectasia; GERD, gastroesophageal reflux disease; group B: patients taking antithrombotic agents who underwent emergency endoscopic hemostasis for non-variceal upper gastrointestinal bleeding for whom antithrombotic medication was transiently discontinued.
